# Neuroimaging biomarkers and CSF sTREM2 levels in Alzheimer’s disease: a longitudinal study

**DOI:** 10.1038/s41598-024-66211-w

**Published:** 2024-07-03

**Authors:** Fardin Nabizadeh, Homa Seyedmirzaei, Shaghayegh Karami

**Affiliations:** 1 School of Medicine, Iran University of Medical Sciences, Tehran, Iran; 2 Alzheimer’s Disease Institute, Tehran, Iran; 3 School of Medicine, Tehran University of Medical Science, Tehran, Iran; 4 Interdisciplinary Neuroscience Research Program (INRP), Tehran University of Medical Sciences, Tehran, Iran

**Keywords:** Alzheimer’s disease, Amyloid βeta, Tau, TREM2, Microglia, Neuroscience, Neurology

## Abstract

Understanding the exact pathophysiological mechanisms underlying the involvement of triggering receptor expressed on myeloid cells 2 (TREM2) related microglia activation is crucial for the development of clinical trials targeting microglia activation at different stages of Alzheimer’s disease (AD). Given the contradictory findings in the literature, it is imperative to investigate the longitudinal alterations in cerebrospinal fluid (CSF) soluble TREM2 (sTREM2) levels as a marker for microglia activation, and its potential association with AD biomarkers, in order to address the current knowledge gap. In this study, we aimed to assess the longitudinal changes in CSF sTREM2 levels within the framework of the A/T/N classification system for AD biomarkers and to explore potential associations with AD pathological features, including the presence of amyloid-beta (Aβ) plaques and tau aggregates. The baseline and longitudinal (any available follow-up visit) CSF sTREM2 levels and processed tau-PET and Aβ-PET data of 1001 subjects were recruited from the ADNI database. The participants were classified into four groups based on the A/T/N framework: A+ /TN+ , A+ /TN− , A− /TN+ , and A− /TN− . Linear regression analyses were conducted to assess the relationship between CSF sTREM2 with cognitive performance, tau and Aβ-PET adjusting for age, gender, education, and APOE ε4 status. Based on our analysis there was a significant difference in baseline and rate of change of CSF sTREM2 between ATN groups. While there was no association between baseline CSF sTREM2 and cognitive performance (ADNI-mem), we found that the rate of change of CSF sTREM2 is significantly associated with cognitive performance in the entire cohort but not the ATN groups. We found that the baseline CSF sTREM2 is significantly associated with baseline tau-PET and Aβ-PET rate of change only in the A+ /TN+ group. A significant association was found between the rate of change of CSF sTREM2 and the tau- and Aβ-PET rate of change only in the A+ /TN− group. Our study suggests that the TREM2-related microglia activation and their relations with AD markers and cognitive performance vary the in presence or absence of Aβ and tau pathology. Furthermore, our findings revealed that a faster increase in the level of CSF sTREM2 might attenuate future Aβ plaque formation and tau aggregate accumulation only in the presence of Aβ pathology.

## Introduction

Alzheimer’s disease (AD) is a neurodegenerative disorder and the most common cause of dementia, identified by the decline in cognitive abilities and functional capacity^1^. AD is characterized by extracellular amyloid-beta (Aβ) plaques (derived from amyloid precursor protein (APP)) and intracellular accumulation of hyperphosphorylated tau protein^2^. Aβ aggregates are observed in brain regions with high metabolic activity, including the neocortex, brainstem, and, ultimately, the cerebellum^3^. Also, tau pathology initially occurs in the locus coeruleus and limbic regions, afterward progressing to the neocortex^4^. However, tau pathology showed a stronger correlation with cognitive function compared to Aβ pathology and demonstrates a notable regional concordance with areas of neurodegeneration^5^.

Aβ is known to have a pivotal role in the pathogenesis of AD as it precedes the occurrence of tau pathology and triggers the conversion of tau protein from its normal state to a toxic one^6^. Also, the presence of toxic tau amplifies the toxicity of Aβ via a feedback mechanism^7^. Recently, there were efforts to provide a biological rather than a syndromal definition of AD based on biomarkers to understand the underlying mechanism and discover interventions that prevent or delay the pathology. The pattern of reduction in Aβ42 along with increased total tau (T-tau) and phosphorylated tau (P-tau) often called the “Alzheimer CSF profile”^1,2,5,8^. Also, there are continuous investigations for alternative biomarkers in order to achieve better diagnostic performance and more reliable measurement of the efficacy of treatments.

As far as the amyloid cascade hypothesis is questioned due to the contradictory findings, it is believed that the disruption in brain cell hemostasis plays a role in the development of AD^9^. As a primary response to brain damage, a large number of studies were conducted on the possible role of microglia in AD pathology^9^. One of the neuroinflammation biomarkers that may be utilized for early detection, prognosis, and therapeutic aims is triggering receptor expressed on myeloid cells 2 (TREM2), which is involved in the conversion of microglia from a hemostatic state to a disease-associated state^10^. The extracellular domain of TREM2 can be cleaved into soluble TREM2 (sTREM2) (a microglial activation biomarker), which is then released into the CSF and plasma^11–13^. CSF sTREM2 levels are affected by genetic mutations, physiological conditions, and underlying disease^14–17^.

Investigating the possible protective or detrimental role of TREM2-related microglia activation in AD yielded contradictory findings^13^. Several studies showed elevated CSF sTREM2 levels in preclinical and early stages of AD, while several investigations have shown no difference or even a reduced level of sTREM2 in AD patients compared to healthy controls^18–24^. Further studies aimed to find the association of TREM2-related microglia activation also showed contradictory results. Several studies revealed that sTREM2 is highly associated with the tau pathology measured in CSF tau, which supports TREM2-related microglia activation as a detrimental factor for tauopathy in AD^19,22,24–26^. Also, another study revealed that TREM2-related microglia activation is associated with Aβ-related tau pathology^25^. However, other studies showed that the CSF sTREM2 levels are associated with reduced Aβ accumulation and, consequently, with lower risk for cognitive decline and neurodegeneration in cognitively normal to AD dementia subjects, implying that TREM2-related microglia activation might be a protective factor on Aβ accumulation^26,27^. Although there is evidence from both animal and clinical research indicating the preventive effect of sTREM2 in alleviating the pathology associated with AD, some studies reported inconsistent results.

Understanding the exact pathophysiology behind the role of TREM2-related microglia activation is crucial for clinical trials targeting microglia activation in different stages of AD. Due to the contradictory findings, it is necessary to investigate the longitudinal changes in CSF sTREM2 as a marker for microglia activation and AD biomarkers to fill the current gap in the evidence. In this study, we aimed to measure the longitudinal changes in CSF sTREM2 level in the A/T/N classification system for AD biomarkers and determine the possible association with AD pathological hallmarks including Aβ plaques and tau aggregates. The A/T/N classification system is a biomarker-based classification published by the National Institute on Aging and the Alzheimer’s Association in 2018. This system consists of three domains: Aβ pathology (A), tau pathology (T), and neurodegeneration (N), which are predictors of AD prognosis^28^.

## Methods and materials

### Participants

This cross-sectional and longitudinal study examines the levels of CSF sTREM2 in a cohort of 1001 participants from the Alzheimer’s disease neuroimaging initiative (ADNI) project. Participants diagnosed with dementia in the baseline or during follow-up were excluded from the analysis. From the dataset, we included participants aged between 55 and 90 years, who had a minimum of 6 years of education, were fluent in either Spanish or English, without any significant neurological disorder other than AD, and had available either baseline or longitudinal CSF sTREM2, Aβ-PET and tau-PET scans. The baseline and longitudinal CSF sTREM2 measurements were obtained from the ADNI database (http://adni.loni.usc.edu). Available data for cross-sectional and longitudinal analysis at each time point detailed in Supplementary 1. The ADNI project, led by principal investigator Michael W Weiner, is a multicenter longitudinal study aimed at the development and validation of biomarkers for subject selection and as surrogate outcome measures in late-onset AD. The study procedures were approved by the institutional review boards (IRB) of all participating centers, and informed consent was obtained from all participants or their surrogates. Additionally, the study was approved by our local IRB (LMU). All methods were performed in accordance with the relevant guidelines and regulations.

### Classification

In accordance with the 2018 NIA-AA “research framework” for Alzheimer’s disease diagnosis^29^, participants from the ADNI project were grouped based on their biomarker profile using the A/T/N scheme^30^. This scheme comprises three biomarker categories: “A” for aggregated Aβ, “T” for aggregated tau, and “N” for neurodegeneration. Each biomarker category was classified as either negative ( −) or positive ( +) depending on the normality or abnormality of the specific biomarkers.

In this research, participants were labeled as “A+ ” if their CSF Aβ1-42 level was below 976.6 pg/ml, “T+ ” if their P-tau181P level exceeded 21.8 pg/ml, and “N+ ” if their T-tau level surpassed 245 pg/ml. To facilitate comparisons, we merged the aggregated tau (T) and neurodegeneration (N) categories. Consequently, TN negative (TN−) was defined as having both aggregated tau (T) and neurodegeneration (N) biomarkers within the normal range (T- and N-, meaning P-tau181P ≤ 21.8 pg/ml and T-tau ≤ 245 pg/ml). Participants were categorized as TN positive (TN+) if either the aggregated tau (T) or neurodegeneration (N) biomarkers were abnormal (T+ or N+ , i.e., P-tau181P > 21.8 pg/ml or T-tau > 245 pg/ml). Only 5.2% of the total participants showed differences between the aggregated T and N biomarker categories^31^.

### CSF sTREM2 measurements

CSF sTREM2 measurements were performed using a validated MSD platform-based assay, as previously reported^21,32^. The CSF sTREM2 measurements utilized in this study are publicly accessible in the ADNI database.

### PET imaging

In the ADNI study, AV45-PET (Aβ) imaging was performed over six 5 min intervals, around 60–90 min after the administration of 370 Mbq radiolabeled F18-AV45 tracer. These six intervals were then aligned and averaged to produce a mean AV45 image. The global AV45 standardized uptake value ratio (SUVR) was computed as the mean value across specific cortical regions, standardized to a composite reference region covering the entire cerebellum and cerebral white matter. This approach is based on a previously outlined procedure that confirmed the consistency of longitudinal AV45 changes using this composite reference region^33^. To evaluate longitudinal alterations in AV45 uptake, we determined the yearly rate of change in global AV45 SUVR values for each participant. Aβ-PET rate of change was measured using a linear mixed effect model since some of the participants had more than two AV45 scans^31^.

Tau-PET imaging was carried out 75 min after the injection of F18-radiolabeled AV1451 tracer, using six 5 min segments. The captured images were aligned and averaged across segments, then normalized for intensity to the inferior cerebellar gray matter, following the methodology described in Maass et al^34^. Specific SUVR scores for regions of interest (ROIs) defined by Braak stage were sourced from the ADNI core and retrieved from the ADNI database. Detailed protocols for these processes can be accessed on the ADNI website and in previous publications^35^. It is noteworthy that Braak stage 2 (specifically the hippocampus) was excluded from the analysis due to documented off-target binding of the AV1451 tracer in this particular area, as indicated in Lemoine et al^36^. Tau-PET rate of change was measured using a linear mixed effect model since some of the participants had more than two AV1451 scans^31^.

### Statistical analysis

All statistical analyses were carried out using the R software. The normality of the data was assessed using the Kolmogorov–Smirnov test. Variables that did not follow a normal distribution were transformed by taking the logarithm (base 10) before further analysis. Baseline sociodemographic data were compared among the ATN groups using analysis of variance (ANOVA), while chi-square tests were utilized for comparing categorical variables such as APOE status and gender. To address multiple comparisons, Bonferroni correction was implemented for all post hoc tests. To evaluate the change in CSF sTREM2 concentrations, tau-PET, and Aβ-PET over time, individual slopes were calculated for each participant using a linear mixed-effects model (LMEM) from the lme4 R package. The LMEM incorporated time (years from baseline) as a fixed factor and subject as a random factor. Subsequently, ANOVA was employed to compare biomarker slopes and baseline levels across the ATN groups. Linear regression models were employed to investigate whether the baseline concentration and rate of change in CSF sTREM2 level could predict ADNI memory performance and PET tau and Aβ burden. All analyses were adjusted for age, gender, education, and APOE ε4 status. Linear regression analyses were conducted to assess the relationship between CSF sTREM2 (baseline and slope) and tau and Aβ-PET (baseline and rate of change) for the entire cohort and within individual ATN groups, adjusting for age, gender, education, and APOE ε4 status. The Bonferroni correction method was applied to mitigate false positives with a significance level set at *P* < 0.05.

### Ethical approval and consent to participate

Since the data in this paper were obtained from the ADNI database (adni.loni.usc.edu), it does not include any research involving human or animal subjects.

## Results

The sociodemographic and clinical data of the included participants are presented in Table 1.

**Table 1:** Demographic and clinical characteristics.

Variable	A− /TN− (n = 246)	A + /TN − (n = 166)	A + /TN + (n = 407)	A-/TN + (n = 182)	*P*-value
Age (years)	71.2 ± 6.8	73.0 ± 7.0	73.7 ± 7.4	74.0 ± 7.6	< 0.001
Female (%)	113 (46%)	53 (32%)	184 (45%)	88 (48%)	0.008
Education (years)	16.2 ± 2.7	16.2 ± 2.8	15.8 ± 2.8	16.0 ± 2.6	0.201
APOEε4 carriers (%)	43 (17%)	77 (36%)	300 (73%)	50 (27%)	< 0.001
MMSE	28.6 ± 1.5	27.6 ± 2.3	25.9 ± 2.8	28.1 ± 2.1	< 0.001
CSF sTREM2 (pg/ml)	3570.3 ± 1748.1	2795.0 ± 1500.4	4376.0 ± 2299.3	5389.8 ± 2359.7	< 0.001
Aβ-PET at baseline	1.02 ± 0.07	1.31 ± 0.20	1.43 ± 0.17	1.05 ± 0.17	< 0.001
Aβ-PET rate of change	0.002 ± 0.010	0.008 ± 0.015	0.012 ± 0.018	0.006 ± 0.012	< 0.001
tau-PET at baseline	1.47 ± 0.13	1.45 ± 0.22	1.97 ± 0.43	1.72 ± 0.17	< 0.001
tau-PET rate of change	0.009 ± 0.056	0.030 ± 0.097	0.074 ± 0.134	0.052 ± 0.070	< 0.001
MCI (%)	147 (60%)	101 (61%)	225 (55%)	111 (61%)	0.112

Data are presented as mean ± standard deviation unless specified otherwise.

*APOEε4* apolipoprotein E genotype (carrying at least one ε4 allele), *CSF sTREM2* cerebrospinal fluid soluble triggering receptor expressed on myeloid cells 2, *MMSE* mini-mental state evaluation, *MCI* mild cognitive impairment, *A* Aβ pathology, *TN* tau neurodegeneration.

### Baseline differences and rate of change of CSF sTREM2 concentrations between the ATN groups

**Figure 1:**
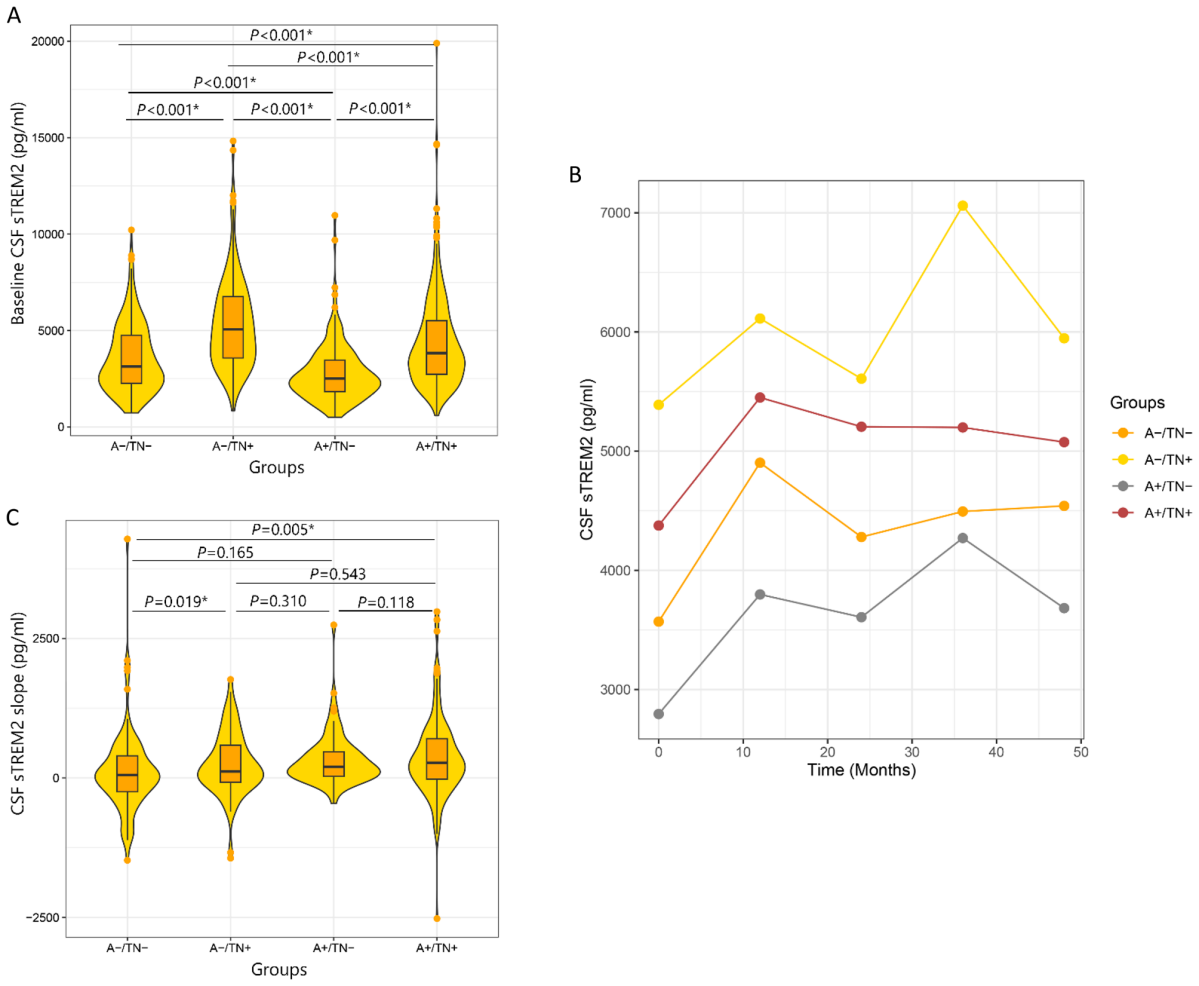
(**A**) Baseline level of CSF sTREM2 were compared between groups and (**B**) longitudinal trajectories of CSF sTREM2 and (**C**) rate of change of CSF sTREM2.

Based on our analysis there was a significant difference in CSF sTREM2 (*P* < 0.001) between ATN groups (Fig. 1). The post hoc pairwise comparisons showed a significant difference between groups for CSF sTREM2 (*P* < 0.001) by increasing concentration with A+ /TN−  < A− /TN−  < A+ /TN+  < A− /TN+ .

We estimated the trajectory of CSF sTREM2 over 4 years of follow-up across the ATN group using linear mixed-effect models adjusted for the effect of age, sex, education, and APOE ε4 (Fig. 1). Further ANOVA analysis revealed that there was a significant difference in the rate of change of CSF sTREM2 (*P* = 0.015) between ATN groups. The Bonferroni post hoc pairwise comparisons showed that the rate of change of CSF sTREM2 was significantly higher in A+ /TN+ compared to A− /TN− (*P* = 0.005) (Fig. 1). Furthermore, the rate of change of CSF sTREM2 was also higher in A− /TN+ compared to A− /TN− (*P* = 0.019).

### Association between cognitive performance and CSF sTREM2 rate of change

**Figure 2:**
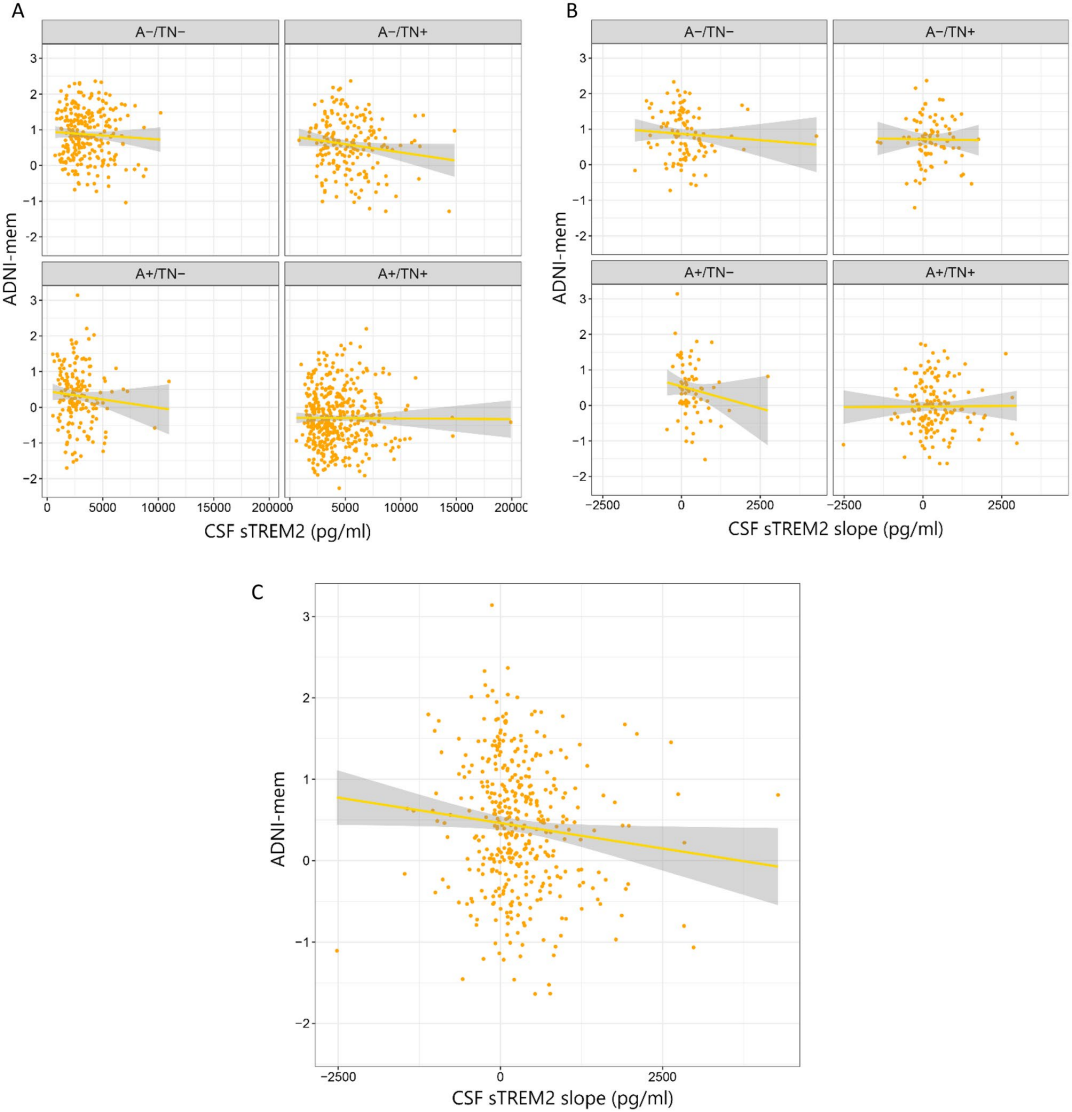
(**A**) Association between cognitive performance (ADNI-mem) and baseline level of CSF sTREM2, (**B**) rate of change of CSF sTREM2 (slope) in ATN groups, and (**C**) all participants.

Using linear regression models adjusted for the effect of age, sex, education, and APOE e4 we found that there was no significant association between the baseline level of CSF sTREM2 and cognitive performance assessed by ADNI-mem across ATN groups (Table 2 and Fig. 2).

**Table 2:** Association of ADNI-mem, tau (flortaucipir) and Aβ (florbetapir) uptake for CSF sTREM2 baseline concentrations and rates of change over time (slope).

Variable	Entire cohort (n = 1001)		A− /TN− (n = 246)		A+ /TN− (n = 166)		A− /TN+ (n = 182)		A+ /TN+ (n = 407)	
	β	*P*-value	β	*P*-value	β	*P*-value	β	*P*-value	β	*P*-value
ADNI-mem										
CSF sTREM2	− 0.028	0.346	0.032	0.606	− 0.029	0.714	− 0.039	0.588	0.009	0.863
CSF sTREM2 slope	− 0.105	**0.042**	− 0.057	0.575	− 0.12	0.323	− 0.013	0.904	− 0.004	0.96
Aβ-PET										
CSF sTREM2	0.026	0.448	0.141	0.059	0.09	0.339	− 0.016	0.849	− 0.045	0.486
CSF sTREM2 slope	**0.178**	** < 0.001**	0.316	0.013	0.157	0.192	0.214	0.098	− 0.015	0.887
Tau-PET										
CSF sTREM2	− 0.067	0.382	− 0.135	0.369	0.039	0.818	0.176	0.313	** − 0.425**	**0.027**
CSF sTREM2 slope	− 0.013	0.899	− 0.01	0.96	− 0.096	0.665	− 0.393	0.062	− 0.224	0.507
Aβ-PET rate of change										
CSF sTREM2	− 0.2	0.684	0.1	0.269	0.126	0.321	0.056	0.605	** − 0.214**	**0.014**
CSF sTREM2 slope	0.063	0.304	− 0.057	0.665	** − 0.309**	**0.038**	0.246	0.078	0.079	0.465
Tau-PET rate of change										
CSF sTREM2	** − 0.218**	**0.011**	** − 0.355**	**0.021**	− 0.074	0.7	− 0.116	0.55	** − 0.476**	**0.028**
CSF sTREM2 slope	− 0.031	0.795	− 0.162	0.42	** − 0.369**	**0.008**	0.218	0.393	− 0.099	0.789

Analyses adjusted for age, sex and APOE e4/PET scan. Baseline cognition was included in the model when predicting ADNI-mem at follow-up.

Significant values are given in bold.

*Aβ* amyloid β, *A*+ */− *amyloid-β positive/negative, *ADNI* Alzheimer’s disease neuroimaging initiative, *ATN* Aβ deposition, tau pathology and neurodegeneration, **β** beta, *sTREM2* soluble triggering receptor expressed on myeloid cell 2, *β* standardized beta, *TN*+ */− *tau/neurodegeneration positive/negative.

We analyzed the association between cognitive performance (ADNI-mem) and rate of change of CSF sTREM2 concentrations using linear regression adjusted for the effect of age, sex, education, and APOE ε4. There was no significant association between cognitive performance (ADNI-mem) and the rate of change of CSF sTREM2 concentrations in ATN groups (Fig. 2). However, using the same model on the total sample size showed that the rate of change of CSF sTREM2 is significantly associated with cognitive performance (β =  − 0.105, *P* = 0.042).

### Association between Tau and Aβ-PET with baseline concentration of CSF sTREM2

**Figure 3:**
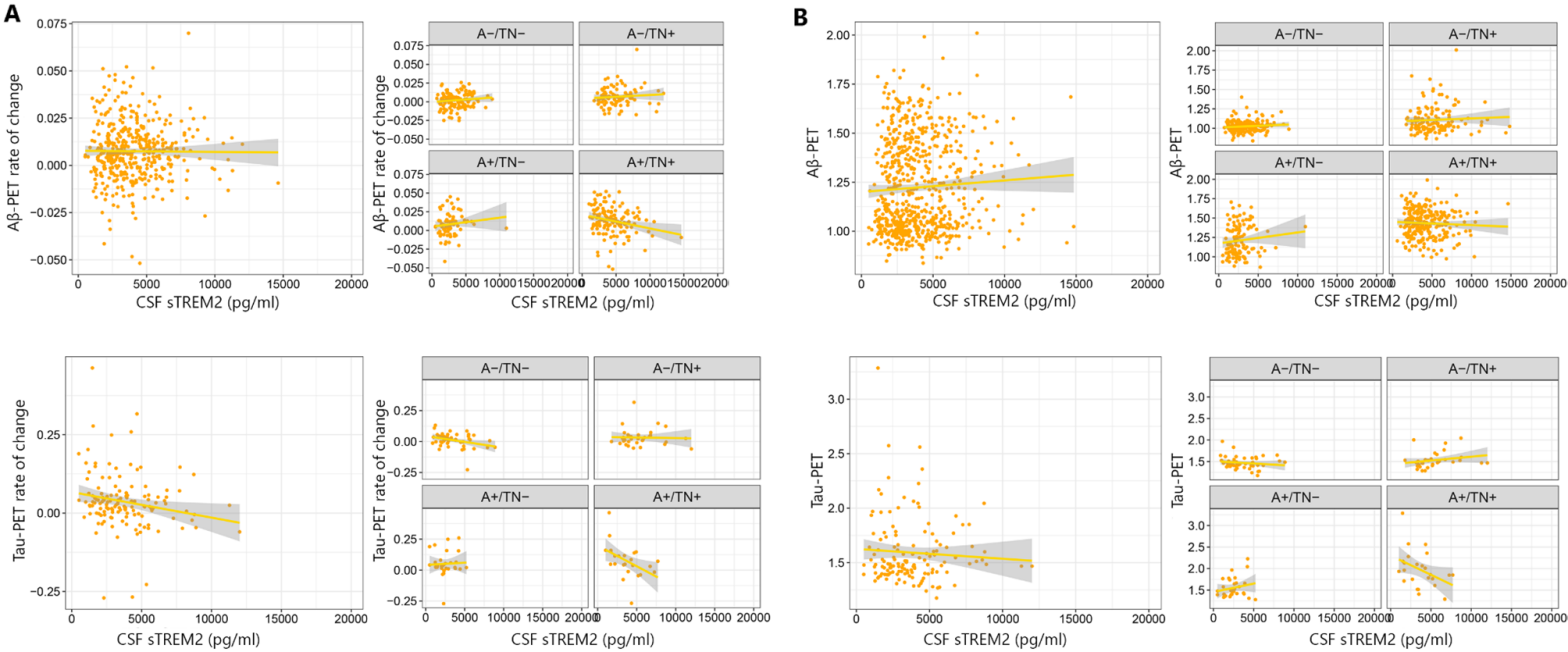
(**A**) Association between baseline CSF sTREM2 and tau and Aβ-PET rate of change, (**B**) association between baseline CSF sTREM2 and baseline tau and Aβ-PET.

In the first step, we investigated the association between baseline neuroimaging findings including tau and Aβ-PET with baseline levels of CSF sTREM2 (Fig. 3). We found that the CSF sTREM2 is significantly associated with baseline tau-PET only in the A+ /TN+ group (Table 2). Next, we aimed to assess the association between longitudinal changes in tau and Aβ-PET (rate of change) with baseline levels of CSF sTREM2. Our analysis revealed that baseline CSF sTREM2 was associated with Aβ-PET rate of change only in the A+ /TN+ group. Furthermore, linear regression analysis showed that baseline CSF sTREM2 was associated with the tau-PET rate of change in A+ /TN+ , A− /TN− , and the entire cohort (Table 2).

### Association between Tau and Aβ-PET with the rate of change of CSF sTREM2

**Figure 4:**
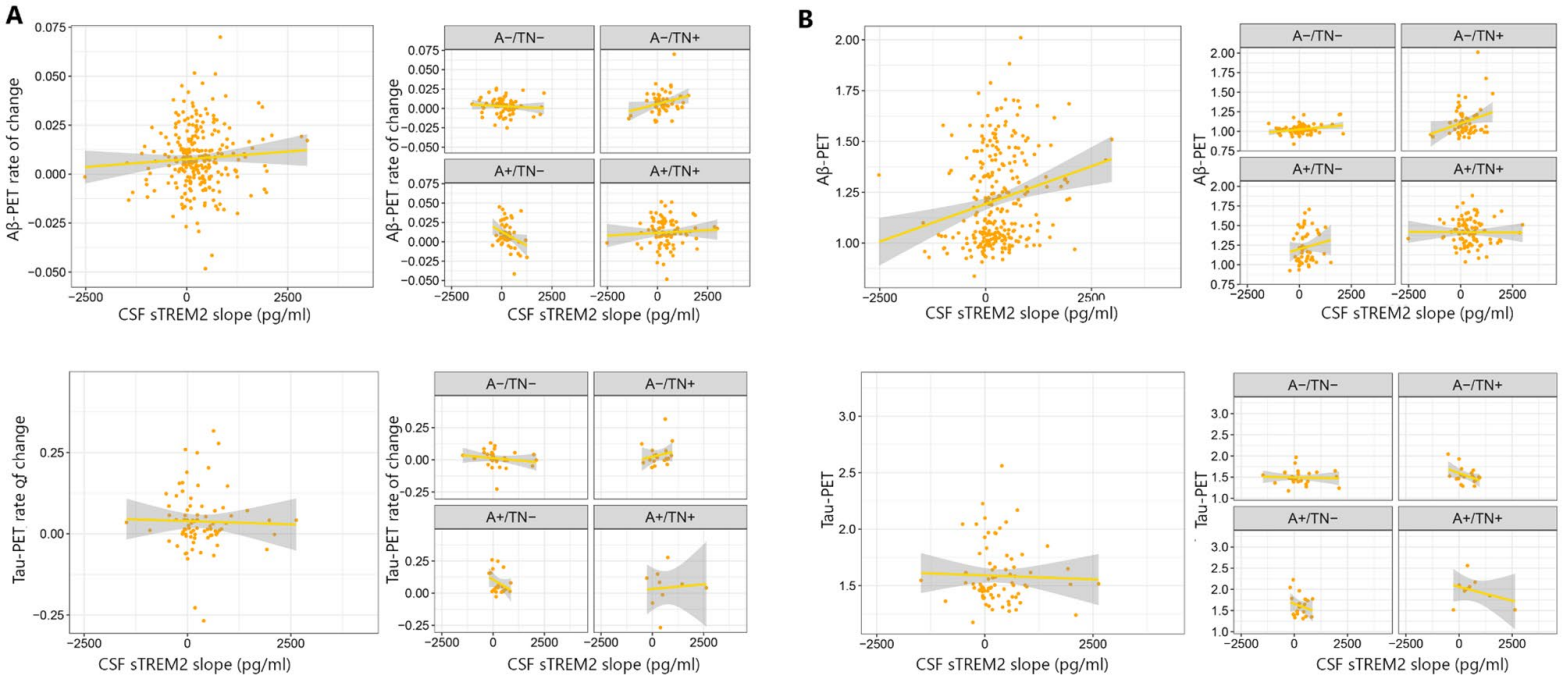
(**A**) Association between rate of change of CSF sTREM2 and tau and Aβ-PET rate of change, (**B**) association between rate of change of CSF sTREM2 and baseline tau and Aβ-PET.

We analyzed if the longitudinal change in CSF sTREM2 (slope) was associated with baseline tau and Aβ-PET using multivariable linear regression analyses. We found that the rate of change of CSF sTREM2 was significantly associated with baseline Aβ-PET in the entire cohort but not ATN groups (Fig. 4). There was no significant association between longitudinal change in CSF biomarkers with baseline tau-PET. We then investigated the possible association between the rate of change of CSF sTREM2 with longitudinal changes in tau and Aβ-PET (rate of change). A significant association was found between the rate of change of CSF sTREM2 and the Aβ-PET rate of change only in the A+ /TN− group. In the end, we found an association between the rate of change of CSF sTREM2 and the tau-PET rate of change only in A+ /TN− .

## Discussion

**Figure 5:**
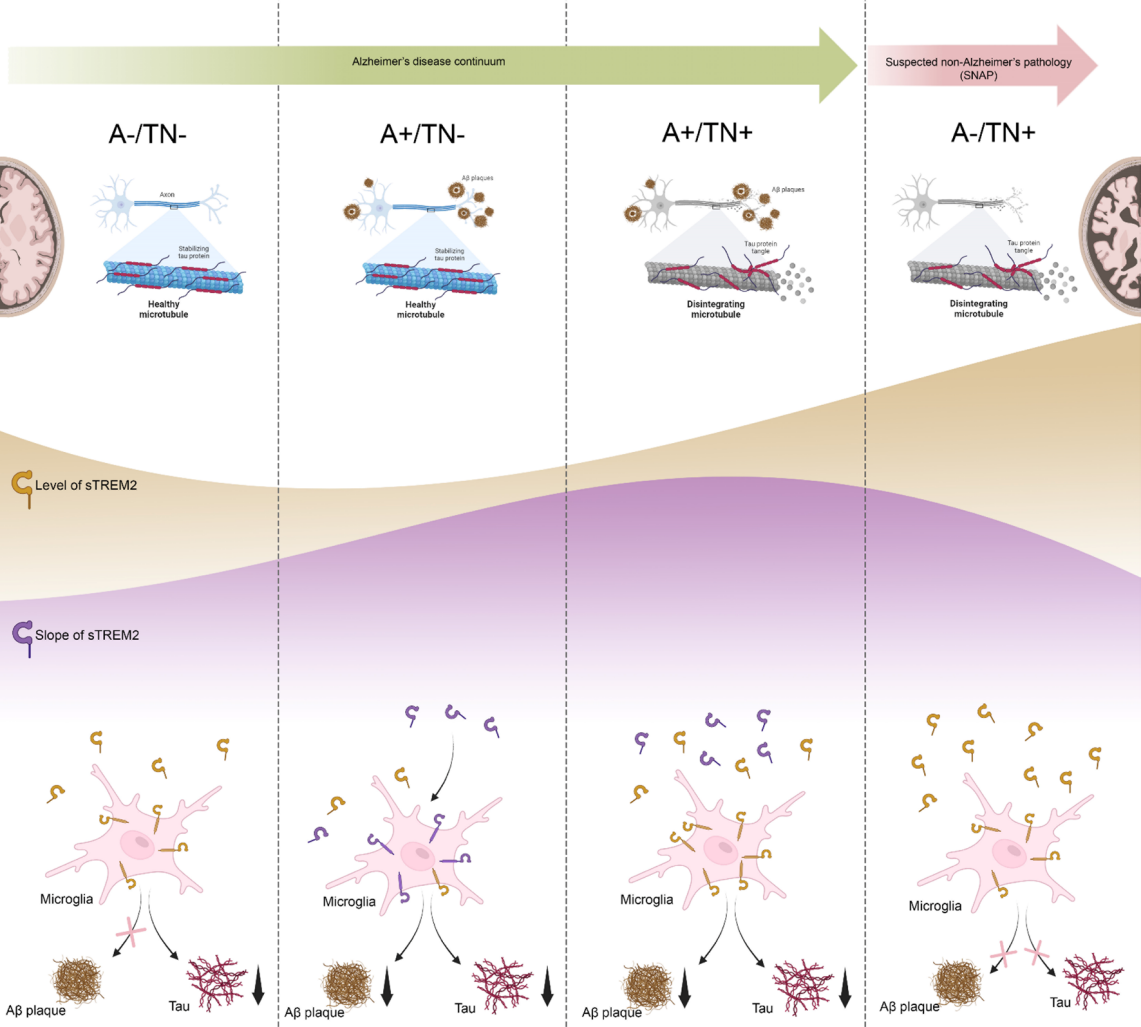
Overview of the CSF sTREM2 levels within the ATN framework.

In the present study, we aimed to portray sTREM2 profiles and their associations with cognition and Aβ and tau-PET cross-sectionally and longitudinally based on the A/T/N framework (Fig. 5). We observed that CSF sTREM2 levels vary among the different ATN groups, as A− /TN+ exhibited the highest levels, followed by A+ /TN+ , A− /TN− , and A+ /TN− , respectively. Following subjects up for 4 years, CSF sTREM2 rises in A+ /TN+ and A− /TN+ were more prominent than those in A− /TN− . Although cognitive decline was not associated with the baseline CSF sTREM2 of any groups, it showed a significant correlation with CSF sTREM2 changes in the total cohort. Investigating the potential neuroimaging associations showed that the baseline sTREM2 is associated with baseline tau-PET and changes of Aβ and tau-PET in A+ /TN+ subjects. Meanwhile, higher rates of change in sTREM2 (slope) during the follow-up were significantly associated with lower rates of change in Aβ and tau-PET in the A+ /TN− group.

Our study highlighted different CSF sTREM2 levels in the AD continuum groups. As previously explained, disparities in the results of studies comparing sTREM2 in AD subjects and normal controls led to the idea of its stage-dependent changes in the AD spectrum^37^. Dividing subjects into groups of CN, preclinical AD, MCI, and AD, the studies of Suárez-Calvet et al. and Gispert et al. reported the highest levels of sTREM2 in MCI than CN, preclinical AD, and AD groups^21,38^. Preclinical AD was defined as asymptomatic people with only decreased CSF Aβ levels (A+ /TN−), while AD and MCI symptomatic people had A+ /TN+ profiles. They classified the participants according to the National Institute on Aging and Alzheimer’s Association (NIA-AA) criteria, which is explained in detail by Jack Jr. et al.^29^.

In contrast to the mentioned studies, we included MCI and CN ADNI subjects based on their clinical manifestations and independently assessed their ATN profiles. As a result, participants could present with or without Aβ pathology, subsequently classifying them in the AD continuum or suspected non-Alzheimer’s pathology (SNAP) according to NIA-AA criteria. In a similar study by Ewers et al., MCI and AD patients with both Aβ and tau pathology had significantly higher CSF sTREM2 than CN people without impaired Aβ and tau profiles^26^. This finding goes in line with our results using the same dataset with a higher sample size, in which baseline sTREM2 levels were greater in participants with both Aβ and tau pathology compared to subjects without any pathology. As we found no association between baseline level of CSF sTREM2 and cross-sectional cognitive performance, the mentioned study revealed that higher baseline CSF sTREM2 concentrations are associated with a slower decline in ADNI-MEM scores over time^26^.

The amyloid cascade hypothesis has been the mainstream explanation of AD pathology for over 30 years^39^. In brief, it suggests that first Aβ peptides may be accumulated around the neurons and form Aβ amyloid fibrils. These accumulated fibrils result in a neurotoxic environment, that can induct tau pathology, leading to neuronal cell death and neurodegeneration^40^. Our study showed that in people with only Aβ pathology before the occurrence of tau neurodegeneration, a higher change of CSF sTREM2 is associated with a lower rate of change in Aβ and tau deposition. However, when people reach a phase with impaired tau/neurodegeneration status regardless of Aβ pathology (A+ /TN+ and A− /TN+), these associations are uncoupled; although sTREM2 rises at a faster speed than ever, its alterations are no longer associated with longitudinal changes in Aβ and tau PET. These findings suggest that a longitudinal increase in TREM2-related microglia activation can be a protective factor for future Aβ plaque formation and tau aggregate accumulation in the preclinical stage of AD.

A rodent study has shown that germline knockout of TREM2 decreases microgliosis around Aβ plaques and accelerates the seeding and spreading of tau aggregates around the neuritic plaque^41^. Meanwhile, the assessment of both rodent AD models and human subjects has shown that when incapable of fully neutralizing tau seeding activity, microglial cells release pathological tau seeds into the extracellular space and deteriorate tau hyperphosphorylation profiles^42^. Considering microglial activity, a double-edged sword, our findings can partially be explained in light of the traditional amyloid cascade hypothesis. We suggest that in the early AD continuum when Aβ pathology has appeared, microglial cells aggregate around plaques and exhibit anti-inflammatory roles to phagocytose Aβ plaques^43^. During this phase, sTREM2 is retained in the plaques and it might be the reason that people with only Aβ and pathology (A+ /TN−) show the lowest baseline levels of sTREM2 in our study. However, long-lasting exposure between microglia and Aβ plaques over-activate these cells and elevates pro-inflammatory cytokines such as interleukin 1-β, interleukin 6, and tumor necrosis factor-alpha, which can, in turn, impair microglial cells phagocytosis activity in a vicious loop^44^. At last, the protective microglial barrier falls apart, sTREM2 levels increase, and phosphorylated tau proteins are released. According to our results, there will be a point in the AD continuum when sTREM2 reaches its highest levels later but its elevations are no longer associated with attenuation in phosphorylated tau, suggesting that pro-inflammatory microglial cells are not protective against the disease pathological changes until late AD.

Based on our cross-sectional assessments, baseline tau PET was negatively associated with baseline CSF sTREM2 in people with both pathological Aβ and tau (A+ /TN+), as opposed to Aβ PET. The correlation between CSF sTREM2 and tau accumulation (in the absence of CSF sTREM2 and Aβ plaques association) has been described repeatedly in other studies^21,25,32,45^. Accordingly, a post-mortem study using immunohistochemistry on the brain of the affected patients aimed to find the relationships among microglial activation, tau, and Aβ pathologies^46^. Their analyses showed that the direct effect of microglial activation on tau was the only significant association in both females and males. These findings might imply that the microglial activity’s relation with tau pathology is more direct than that with Aβ pathology in people with impaired Aβ and tau profiles^46^. Another study by Li et al. investigated the CSF sTREM2 levels and AD core biomarkers^47^. Based on their findings CSF sTREM2 level was not correlated with the baseline or longitudinal scale and neuroimaging result changes, and could not predict clinical conversion. However, another study demonstrated that in participants with positive Aβ deposition, higher CSF sTREM2 levels were associated with a decreased risk of MCI-to-AD conversion^48^. Using a longitudinal approach we observed that in subjects with abnormal Aβ plaques, a higher rate of change in sTREM2 can be a protective factor for both future Aβ and tau pathology. Considering the contradictory findings of previous studies and suggested stage-dependent associations, it is crucial to deeply investigate the possible longitudinal associations between TREM2-related microglia activation and AD pathological hallmarks to identify new therapeutic targets. Based on our findings, disease-modifying treatments targeting TREM2 could be effective in preventing future AD pathological changes when the patients are in early stages and have abnormal Aβ levels.

We observed a significant association between CSF sTREM2 and cognitive decline in the whole sample. In a similar study by Ewers et al., it turned out that higher baseline CSF sTREM2 concentrations were associated with a slower decline in ADNI-mem scores over time in MCI and AD people with pathological Aβ and tau^26^. Recruiting autosomal-dominant AD patients and their pre-symptomatic family members has also yielded the findings that an augmented annual rate of increase in sTREM2 is strongly correlated with a decreased annual rate of cognitive decline in pre-symptomatic carriers^49^. All of these findings might imply a potential beneficial effect of TREM2-related microglia activation concentrations on cognition; however, different samples, ATN biomarkers, and cognitive assessments make it hard to reliably generalize the results.

Another finding of our study was the lack of association between CSF sTREM2 and AD pathology hallmarks in participants with tau neurodegeneration and normal Aβ deposition which is called SNAP. It can be concluded that the TREM2-related microglia activity might have a different role in non-AD pathophysiology and shows the specific role of TREM2 on tau and Aβ deposition only when there is an AD-related pathology pattern.

The strength of our study includes a large sample size, both cross-sectional and longitudinal analyses, and the use of PET findings alongside cognitive assessments. Due to the contradictory findings of previous studies, it was necessary to more deeply investigate the stage-dependent role of TREM2-related microglia activation in AD development. However, our study faces some limitations too. First, as we intended to evaluate the AD continuum in its early phases, we only included CN and MCI people and excluded those with dementia. Second, CSF sTREM2 levels may not be a direct measure of microglial activity. The α disintegrin and metalloproteinase 10 (ADAM 10), the TREM2 cleaver, can also release sTREM2 into the interstitial fluid in the brain and affect the results. Third, we considered tau pathology and neurodegeneration as a coupled phenotype, which could have been separately assessed. Note that among the participants with available data, there were less than ten people with unmatched T and N profiles compared to the included 1001 participants. Finally, ethnicity has been reported to influence microglial activation markers^50^. Subjects of ADNI are generally recruited from American and Canadian centers, which limits the race variations in the sample.

## Conclusion

Our study suggests that the TREM2-related microglia activation and their relations with AD markers and cognitive performance vary in the presence or absence of Aβ and tau pathology. Furthermore, our findings revealed that a faster increase in the level of CSF sTREM2 might attenuate future Aβ plaque formation and tau aggregate accumulation only in the presence of Aβ pathology. These results can have direct therapeutic relevance, as there are currently clinical trials targeting TREM2-based microglial functions through monoclonal antibodies such as AL002 in the AD continuum. (Clinical trials: NCT04592874 and NCT05744401). The results of these trials can better shed light on the clinical significance of TREM2 in AD. Future studies including other microglial activation markers, ethnically enriched samples, and recruiting people with both sporadic and genetic AD continuum diseases can better replicate and complete the results of our study.

## Supplementary Information

Supplementary Table 1.

## Data Availability

The datasets analyzed during the current study are available upon request with no restriction. Please contact Dr. Fardin Nabizadeh (fardinnabizade1378@gmail.com) to access data.
