# Author Correction: Neuroimaging biomarkers and CSF sTREM2 levels in Alzheimer’s disease: a longitudinal study

**DOI:** 10.1038/s41598-024-77478-4

**Published:** 2024-11-08

**Authors:** Fardin Nabizadeh, Homa Seyedmirzaei, Shaghayegh Karami

**Affiliations:** 1 School of Medicine, Iran University of Medical Sciences, Tehran, Iran; 2 Alzheimer’s Disease Institute, Tehran, Iran; 3 School of Medicine, Tehran University of Medical Science, Tehran, Iran; 4 Interdisciplinary Neuroscience Research Program (INRP), Tehran University of Medical Sciences, Tehran, Iran

Correction to: *Scientific Reports* 10.1038/s41598-024-66211-w, published online 03 July 2024

The original version of this Article contained errors. In the Introduction, Reference 27 was incorrectly cited.

“Also, another study revealed that TREM2-related microglia activation is associated with Aβ-related tau pathology^27^.”

now reads:

“Also, another study revealed that TREM2-related microglia activation is associated with Aβ-related tau pathology^25^.”

Additionally, Reference 16 and 25 were incorrectly cited in the same paragraph.

 “However, other studies showed that the CSF sTREM2 levels are associated with reduced Aβ accumulation and, consequently, with lower risk for cognitive decline and neurodegeneration in cognitively normal to AD dementia subjects, implying that TREM2-related microglia activation might be a protective factor on Aβ accumulation^16,25,26,28^.”

now reads:

“However, other studies showed that the CSF sTREM2 levels are associated with reduced Aβ accumulation and, consequently, with lower risk for cognitive decline and neurodegeneration in cognitively normal to AD dementia subjects, implying that TREM2-related microglia activation might be a protective factor on Aβ accumulation^26,27^.”

As a result, References 27–32 have been renumbered.

Furthermore, Reference 42 was incorrectly stated as:

Hopp, S. C. *et al*. The role of microglia in processing and spreading of bioactive tau seeds in Alzheimer’s disease. *J. Neuroinflamm.* **15**, 269 (2018).

The correct Reference 42 is listed below:

Gratuze M. *et al*. Activated microglia mitigate Aβ-associated tau seeding and spreading. *J. Exp. Med.* **218**(8), e20210542 10.1084/jem.20210542 (2021).

The original Article has been corrected.

