# Supplementary material for: Neuroimaging biomarkers and CSF sTREM2 levels in Alzheimer’s disease: a longitudinal study
Source: Sci Rep. 2024 Jul 3;14:15318. doi: 10.1038/s41598-024-66211-w (PMC11222555; doi:10.1038/s41598-024-66211-w)
Supplement: Supplementary file 1 — Supplementary Table 1. [file 41598_2024_66211_MOESM1_ESM.docx]

| Supplementary 1. Number of participants with available CSF sTREM2 and brain PET at each time points | | | | |
| --- | --- | --- | --- | --- |
| Variable | A-/TN- (n=246) | A+/TN- (n=166) | A+/TN+ (n=407) | A-/TN+ (n=182) |
| CSF sTREM2 at baseline | 246 | 166 | 407 | 182 |
| CSF sTREM2 at 12 months | 34 | 29 | 55 | 32 |
| CSF sTREM2 at 24 months | 85 | 61 | 128 | 74 |
| CSF sTREM2 at 36 months | 22 | 21 | 36 | 20 |
| CSF sTREM2 at 48 months | 52 | 44 | 85 | 54 |
| Aβ-PET at baseline | 134 | 111 | 278 | 116 |
| Aβ-PET at 24 months | 91 | 78 | 199 | 71 |
| Aβ-PET at 48 months | 45 | 34 | 141 | 63 |
| tau-PET at baseline | 178 | 98 | 173 | 97 |
| tau-PET at 24 months | 76 | 55 | 108 | 43 |
| tau-PET at 48 months | 44 | 32 | 86 | 33 |
| Abbreviations: Aβ, Amyloid Beta; A, Aβ pathology; TN, Tau neurodegeneration; CSF, cerebrospinal fluid; sTREM2, soluble Triggering Receptor Expressed on Myeloid cells 2 | | | | |
